# Reducing default mode network connectivity with mindfulness-based fMRI neurofeedback: a pilot study among adolescents with affective disorder history

**DOI:** 10.1038/s41380-023-02032-z

**Published:** 2023-03-30

**Authors:** Jiahe Zhang, Jovicarole Raya, Francesca Morfini, Zoi Urban, David Pagliaccio, Anastasia Yendiki, Randy P. Auerbach, Clemens C. C. Bauer, Susan Whitfield-Gabrieli

**Affiliations:** 1 Department of Psychology, Northeastern University, Boston, MA 02115 USA; 2 Department of Psychiatry, Columbia University, New York, NY 10032 USA; 3 New York State Psychiatric Institute, New York, NY 10032 USA; 4 Athinoula A. Martinos Center for Biomedical Imaging, Massachusetts General Hospital and Harvard Medical School, Boston, MA 02129 USA; 5 Division of Clinical Developmental Neuroscience, Sackler Institute, New York, NY 10032 USA; 6 Department of Brain and Cognitive Sciences and McGovern Institute for Brain Research, Massachusetts Institute of Technology, Cambridge, MA 02139 USA

**Keywords:** Depression, Neuroscience

## Abstract

Adolescents experience alarmingly high rates of major depressive disorder (MDD), however, gold-standard treatments are only effective for ~50% of youth. Accordingly, there is a critical need to develop novel interventions, particularly ones that target neural mechanisms believed to potentiate depressive symptoms. Directly addressing this gap, we developed mindfulness-based fMRI neurofeedback (mbNF) for adolescents that aims to reduce default mode network (DMN) hyperconnectivity, which has been implicated in the onset and maintenance of MDD. In this proof-of-concept study, adolescents (*n* = 9) with a lifetime history of depression and/or anxiety were administered clinical interviews and self-report questionnaires, and each participant’s DMN and central executive network (CEN) were personalized using a resting state fMRI localizer. After the localizer scan, adolescents completed a brief mindfulness training followed by a mbNF session in the scanner wherein they were instructed to volitionally reduce DMN relative to CEN activation by practicing mindfulness meditation. Several promising findings emerged. First, mbNF successfully engaged the target brain state during neurofeedback; participants spent more time in the target state with DMN activation lower than CEN activation. Second, in each of the nine adolescents, mbNF led to significantly reduced within-DMN connectivity, which correlated with post-mbNF increases in state mindfulness. Last, a reduction of within-DMN connectivity mediated the association between better mbNF performance and increased state mindfulness. These findings demonstrate that personalized mbNF can effectively and non-invasively modulate the intrinsic networks associated with the emergence and persistence of depressive symptoms during adolescence.

## Introduction

In the United States, major depressive disorder (MDD) results in ~$200 billion of lost productivity and health care expenses annually [1], and rates among adolescents are alarmingly high [2]. Gold-standard treatments for depression are only effective for ~50% of youth [3], underscoring the critical need to develop novel treatments to improve clinical outcomes.

At the neural systems level, MDD is characterized by elevated resting state connectivity within the default mode network (DMN), which includes core midline hubs in the subgenual anterior cingulate cortex (sgACC), medial prefrontal cortex (MPFC), and posterior cingulate cortex (PCC) [4, 5]. Although the sgACC is not typically considered a DMN node in ICA-based analyses (likely due to its low signal-to-noise ratio compared to other major DMN nodes at 3 Tesla), seed-based connectivity analyses have consistently placed the sgACC within canonical DMN topography (e.g., [6, 7]). DMN hyperconnectivity, especially sgACC hyperconnectivity, is associated with symptom severity in depressed individuals [8, 9] and characterizes children with elevated familial risk for depression [10]. The DMN is thought to facilitate patterns of depressogenic, self-referential processing and a heightened focus on distressing emotional states [4, 11–13]. In MDD, it is theorized that dysregulation of the DMN by top-down control networks, such as the central executive network (CEN) [14, 15], also contributes to heightened self-focus. Accordingly, hyperconnectivity within the DMN has been linked to rumination (i.e., the tendency to perseverate about one’s symptoms), a common trait that contributes to depression onset, maintenance, and recurrence [16–18] as well as cognitive therapy non-response and relapse [17, 19].

As DMN connectivity is a promising biomarker of MDD [9, 20], new interventions targeting DMN have been explored. For example, transcranial magnetic stimulation (TMS) targeting the dorsolateral prefrontal cortex (i.e., a CEN node that is anticorrelated with DMN) normalizes DMN connectivity and improves depressive symptoms in adults [21]. Interestingly, mindfulness meditation also can lead to decreased DMN activity [22–26] and connectivity [27], and importantly, improves depression treatment outcomes [28–31]. Although research has demonstrated that adolescents can apply mindfulness practices to reduce depression symptoms [32, 33], certain depressive symptoms (e.g., inattention, lack of energy, apathy) may prevent adolescents from more successfully integrating and applying mindfulness strategies in daily life.

To facilitate the acquisition and utilization of mindfulness strategies, we recently developed a novel mindfulness-based fMRI neurofeedback (mbNF; [34]) approach, which is a non-invasive technique that allows people to track and modulate brain function. To date, neurofeedback studies in depression have frequently involved mood-related tasks, such as negative emotion induction or valenced autobiographical memory recall (see review in [35]). By contrast, our mbNF aims to reduce DMN connectivity given associations with mindfulness and MDD. In this 15-min neurofeedback paradigm, people observe a schematic visual representation of their brain activity and practice mindfulness to volitionally reduce DMN relative to CEN activation. We previously demonstrated that mbNF reduced DMN connectivity in adults with schizophrenia and led to symptom reduction post-intervention [36]. As this mbNF method is non-invasive and optimizes the implementation of mindfulness to reduce DMN connectivity, it has enormous potential to facilitate skill acquisition outside of the scanner.

Building on our prior fMRI neurofeedback research [36, 37], we tested the feasibility of mbNF in adolescents with a history of affective disorders in this non-randomized, single-arm, proof-of-concept study. First, we tested whether adolescents would spend more time in the target state, characterized by lower DMN than CEN activation. Second, we tested whether mbNF leads to reduced DMN connectivity and associated increases in state mindfulness. Third, we tested whether reduced DMN connectivity accounted for the association between successful neurofeedback and increased state mindfulness.

## Methods and materials

### Participants and procedure

Adolescents (*n* = 9; 18.8 ± 0.7 years; 17–19 years; 66.7% females) who previously completed scans for the Boston Adolescent Neuroimaging of Depression and Anxiety Human Connectome project (BANDA; [38, 39]) were re-contacted, screened, and enrolled in this proof-of-concept study. Sociodemographic and clinical characteristics are summarized in Table 1; all participants reported a lifetime history of MDD and/or anxiety disorders. Three participants exhibited current diagnoses and are highlighted in figures where applicable; two reported clinical levels of MDD and anxiety disorders and one reported anxiety disorder only. Neurofeedback performance or post-neurofeedback changes did not differ based on the current vs. lifetime presence of diagnoses.

**Table 1:** Sociodemographic and clinical information (*n* = 9).

Category	
Sociodemographic	
Age (mean/SD)	18.8 (0.7)
Sex: female (*n*/%)	6 (66.7%)
Pubertal status: stage (mean/SD)	4.28 (0.6)
Handedness: right (*n*/%)	9 (100.0%)
Race: White (*n*/%)	8 (88.9%)
IQ (mean/SD)	115.1 (15.3)
Current symptoms (mean/SD)	
Depression symptoms (MFQ)	17.4 (13.7)
General anxiety symptoms (RCADS)	3.7 (2.7)
Social anxiety symptoms (RCADS)	13.1 (6.5)
Current psychiatric disorders (*n*/%)	
Comorbid depression and anxiety disorders	2 (22.2%)
Anxiety disorders (without depression)	1 (11.1%)
Any comorbidity (beyond primary depression and/or anxiety)	3 (33.3%)
ADHD	2 (22.2%)
Bulimia	0 (0%)
OCD or related disorders	1 (11.1%)
Oppositional defiant disorder	0 (0%)
Lifetime psychiatric disorders (*n*/%)	
Comorbid depression and anxiety disorder	5 (55.5%)
Anxiety disorders	9 (100.0%)
Any comorbidity (beyond primary depression and/or anxiety)	5 (55.5%)
ADHD	4 (44.4%)
Bulimia	1 (11.1%)
OCD or related disorders	3 (33.3%)
Oppositional defiant disorder	1 (11.1%)
Current medication (*n*/%)	
Any psychiatric medication	6 (66.7%)
Antidepressant medication	6 (66.7%)
ADHD medication	3 (33.3%)

Anxiety disorders = generalized anxiety disorder (*n*_current_ = 1, *n*_lifetime_ = 3), social phobia (*n*_current_ = 2, *n*_lifetime_ = 6), separation anxiety (*n*_current_ = 0, *n*_lifetime_ = 1), and specific phobia (*n*_current_ = 0, *n*_lifetime_ = 1); OCD or related disorders = OCD (*n*_current_ = 1, *n*_lifetime_ = 1), excoriation (*n*_current_ = 0, *n*_lifetime_ = 1), trichotillomania (*n*_current_ = 0, *n*_lifetime_ = 1).

*ADHD* attention-deficit/hyperactivity disorder, *MFQ* Mood and Feelings Questionnaire, *OCD* obsessive-compulsive disorder, *RCADS* Revised Child Anxiety and Depression Scale.

For Session 1, which was a follow-up to the BANDA protocol, study procedures were approved through the Mass General Brigham IRB. At the baseline visit, participants were administered a clinical interview and self-report assessments of depressive and anxiety symptoms. Then, each participant completed a localizer MRI session at the Athinoula A. Martinos Center for Biomedical Imaging. At the end of Session 1, participants were provided with information for Session 2 and interested participants were enrolled. Session 2 procedures were approved by the Northeastern University Institutional Review Board, and typically occurred within 2–3 weeks of Session 1. Participants underwent mindfulness meditation training (15 min), completed a neurofeedback MRI session at the Northeastern University Biomedical Imaging Center (1 h), and completed pre- and post-scan state mindfulness assessments (10 min). With transitions and other tasks/assessments not related to the current analyses, Session 2 typically lasted 2.5 h. Informed consent was obtained from all subjects for both sessions of the study.

### Session 1

#### Clinical assessments

Participants were administered the Kiddie Schedule for Affective Disorders and Schizophrenia Present and Lifetime Version (KSADS; [40]) to provide an assessment of psychiatric disorders as of their most recent study visit, which had occurred 2–3 years prior. Participants also completed the 33-item Mood and Feelings Questionnaire (MFQ; [41]) to assess depression symptom severity. Total scores range between 0–66, with higher scores indicating more severe depression symptoms. Participants also completed the Revised Child Anxiety and Depression Scale (RCADS; [42]). The primary subscales of interest characterize general anxiety and social anxiety symptoms.

#### Functional localizer

MRI data were acquired on a Siemens Prisma scanner with a 64-channel, phased-array head coil (Siemens Healthcare, Erlangen, Germany), including: a T1-weighted MPRAGE structural scan [0.8 mm isotropic voxel size, 208 slices, field-of-view (FOV) = 256 × 240 × 167 mm, repetition time (TR) = 2400 ms, echo time (TE) = 2.18 ms, flip angle (FA) = 8°] and two resting state fMRI scans (rs-fMRI: 5 min 46 s each, eyes open with fixation, multiband acceleration factor = 8, 2 mm isotropic voxels, 72 slices, FOV = 208 × 208 × 144 mm, TR = 800 ms, TE = 37 ms, FA = 52°) to identify participant-specific DMN and CEN maps.

Preprocessing of rs-fMRI data was performed in FSL 6.0 [43] including: motion correction, brain extraction, co-registration, smoothing and bandpass filtering (see more details in [36]). We performed an independent components analysis (ICA) on concatenated preprocessed functional scans using Melodic ICA v3.14 [1] with dimensionality estimation using the Laplace approximation to the Bayesian evidence of the model. Each of the ~30 spatiotemporal components were statistically compared to atlas spatial maps of the DMN and CEN derived from rs-fMRI of ~1000 participants [2] using FSL’s “fslcc” tool and we selected the ICA components that yielded the highest spatial correlation for each participant. ICA components were thresholded to select the upper 10% of voxel loadings and binarized to obtain participant-specific DMN and CEN masks for neurofeedback in Session 2. Visual inspection was performed, and all selected components were determined to be satisfactory in covering canonical DMN (2436.11 ± 328.78 voxels) and CEN (2315.00 ± 105.09 voxels) brain regions [44] (Fig. S1). There was no significant difference between the number of voxels in DMN vs. CEN masks (*p* > 0.05, two-tailed paired-sample *t*-test).

### Session 2

In Session 2, participants completed: pre-mbNF state mindfulness assessment, mindfulness training, structural scan, pre-mbNF rs-fMRI, mbNF, post-mbNF rs-fMRI, post-mbNF state mindfulness assessment.

#### State mindfulness

Participants completed the State Mindfulness Scale (SMS; [45]) by indicating on a 5-point scale their perceived level of awareness and attention to their present experience during the last 15 min. The SMS was scored both as a sum of all 21 items (ranges 0–105) as well as two subscales assessing 15 items on mindfulness of the mind (i.e., thoughts and emotions; ranges 0–75) and 6 items on mindfulness of the body (i.e., movement and physical sensations; ranges 0–30).

#### Mindfulness training

We trained participants on a mindfulness technique called “mental noting”. Mental noting is a major component of Vipassana [46] and consists of the factors “concentration” and “observing sensory experience.” The experimenter explained that mental noting entails being aware of the sensory experience without engaging in or dwelling on the details of the content; in other words, one would “note” the sensory modality (e.g., “hearing,” “seeing,” “feeling”) at the forefront of their awareness and then let it go after it has been noted. The experimenter also introduced the concept of an “anchor”, or a sensory experience to which one could easily switch their attention, such as breathing. Participants were encouraged to use their personal “anchors” when they noted consecutive “thinking” (i.e., rumination). The experimenter demonstrated noting out loud by verbalizing the predominant sensory modality approximately once per second. Participants were then asked to practice mental noting out loud to demonstrate the ability to describe sensory awareness without engaging in the content and stop consecutive “thinking”.

To assess the effectiveness of mindfulness training, participants listened to audio recordings of brief stories before and after training. This included five stories describing everyday, mundane characters and events recorded in neutral tone. Each story lasted about 30 s and included 20 unique details. For example, the sentence “Grandpa owns a garden” includes 3 unique details (i.e., “grandpa,” “owns,” “garden”). Immediately before training (i.e., baseline), they listened to one story and were asked to freely recall as many details as possible. After training, we asked participants to practice mental noting while we played stories (i.e., introducing salient auditory stimulus). The number of stories ranged between 2–4 and we stopped either after the participant was comfortable at the practice or after all remaining 4 stories were played. Compared to the baseline test when participants fully attended to story playback, usage of noting strategy during playback led to a significantly reduced number of details recalled [*t*(8) = −16.20, *p* < 0.001, two-tailed paired-sample *t*-test], indicating that participants were successfully engaging the noting strategy and not retaining the details of the story.

#### mbNF setup

We used multivariate and univariate real-time functional imaging (MURFI), an open source software package to support rs-fMRI neurofeedback. Detailed user manual can be found in [34] and online (https://github.com/cccbauer/MURFI-user-manual). The neurofeedback system is a network of TCP/IP-connected computers including the Siemens MRI scanner, a high-performance Linux laptop running MURFI, and a Windows 10 laptop running stimulus presentation using PsychoPy [47]. During neurofeedback scans, echo-planar imaging (EPI) volumes are continuously reconstructed and transferred from the scanner to the MURFI computer via the TCP/IP connection.

#### mbNF operationalization

**Fig. 1:**
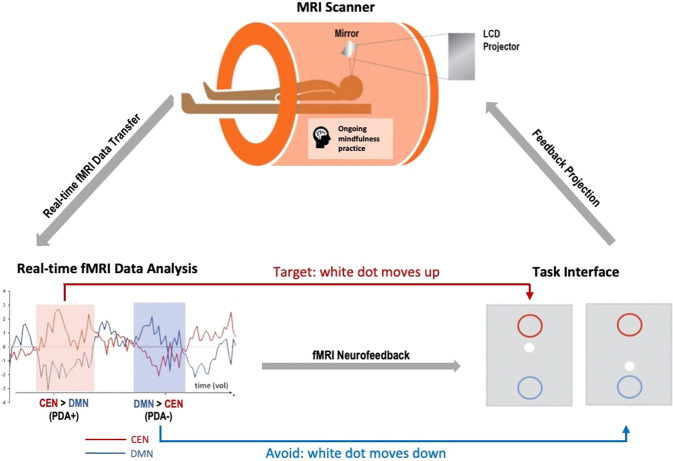
Mindfulness-based neurofeedback (mbNF). During mbNF, participants were instructed to practice mindfulness to move the white dot on the screen up into the red circle. The movement of the white dot was dependent on a real-time analysis of the fMRI data that computed the difference in personalized DMN and CEN activations. When DMN activation is lower than CEN activation, the white dot moves up; when DMN activation is higher than CEN activation, the white dot moves down.

Neurofeedback is a type of biofeedback that provides feedback signal(s) (e.g., visual) based on activation in target brain regions to teach participants to self-modulate brain activation. During neurofeedback, we first displayed a centered crosshair for a 30-s baseline and instructed participants to rest. Then, a continuous 2-min block started with three non-overlapping display items aligned on a vertical axis of a gray screen: a central white dot (*x* = 0, *y* = 0, radius = 12 pixels), a red circle above (*x* = 0, *y* = 1, radius = 56 pixels) and a blue circle below (*x* = 0, *y* = −1, radius = 56 pixels) (Fig. 1). The distance between the centers of the red and blue circles was 472 pixels. The visual feedback was displayed by movements of the white dot. To achieve the visual feedback, MURFI used all data acquired to fit an incremental general linear model (GLM; [48]), where linear trend nuisance signals were discounted and scaled activation estimates were computed for each voxel within each network-of-interest (NOI) (i.e., binarized DMN/CEN mask). It then combined activation across all NOI voxels using the weighted average method. The resulting activation estimate, in units of standard deviations from baseline, per NOI, was immediately sent to the stimulus computer where PsychoPy computed a positive diametric positivity (PDA) metric following the formula described in [27, 36]: ${{{{{\rm{PDA}}}}}} = \widetilde {{{{{{\rm{NOI}}}}}}(\alpha ){{{{{\rm{activation}}}}}}\;{{{{{\rm{estimate}}}}}}} - \widetilde {{{{{{\rm{NOI}}}}}}(\beta ){{{{{\rm{activation}}}}}}\;{{{{{\rm{estimate}}}}}}}$. This PDA metric is based on the hypothesis that there is a causal neural mechanism by which the CEN downregulates the DMN [49]. For each TR, the newly calculated PDA value was added to the *y*-value from the previous TR and the updated *y*-value was rendered as the new position of the white dot. Visually, the white dot would move upwards with positive PDA values and downwards with negative PDA values. The time delay between collection of a complete EPI volume and its associated position update was 30.5 s, and the position updated every TR. Once a participant had accumulated 5 TRs where the white dot was within or beyond a circle, the radius of the corresponding circle shrank by 10% (to titrate difficulty based on performance), and the white dot was repositioned to *y* = 0. The circle would only shrink up to 5 times during one single neurofeedback run.

#### mbNF procedure

There were five back-to-back neurofeedback runs (2.5 min/run). Participants were instructed to move the white dot into the red circle by performing mental noting. Participants were instructed that upward movement of the dot was associated with effective mental noting performance and downward movement with ineffective mental noting, such as self-related processing and mind-wandering. This instruction was provided so that participants can anchor their subjective experience of engaging in mental noting to the observed movements of the white dot. Participants were aware that sustained positions within the red or blue circle (or beyond) would shrink the corresponding circle and return the white dot to the center. After each feedback run, we confirmed whether participants had used mental noting during neurofeedback.

### Session 2 MRI acquisition, preprocessing and data analytic overview

#### MRI acquisition

A structural scan was acquired using a T1-weighted MPRAGE pulse sequence (1 mm isotropic voxel size, 176 slices, FOV = 256 × 256 × 176 mm, TR = 2530 ms, TE = 46 ms, FA = 7°). For functional images, including during mbNF, the BOLD signal was measured using a T2* weighted gradient-echo, EPI pulse sequence (2 mm isotropic voxels, 68 slices, 10% gap, FOV = 256 × 256 × 149.4 mm, TR = 1200 ms, TE = 30 ms, FA = 72°). Each neurofeedback run lasted 2 min and 30 s. Immediately before and after mbNF, two rs-fMRI scans (5 min each, eyes open without fixation) were acquired.

#### MRI preprocessing

Preprocessing was performed using fMRIPrep 21.0.0 [50], which is based on Nipype 1.6.1 [51]. In short, preprocessing included realignment, co-registration, normalization, susceptibility distortion correction, segmentation of gray matter (GM), white matter (WM), cerebrospinal fluid (CSF), skull stripping, and confounds extraction (for details see Supplementary). Visual quality control was performed on each preprocessed run.

Preprocessed data and confound time series were imported into the CONN Toolbox v20.b [52] where outlier identification was performed with the Artifact Detection Tools (ART, www.nitrc.org/projects/artifact_detect). Volumes with global signal *z* > 5 or framewise displacement >0.9 mm compared to the previous frame were flagged as outliers. Relatively lenient thresholds were used to retain data given the small sample size. Comparable results were noted with 8 participants at more stringent thresholds (global signal *z* > 3 and framewise displacement >0.5 mm; see Fig. S2). In addition, in-scanner mean motion was defined as the mean framewise displacement [43] and calculated separately for pre- and post-mbNF runs. Rs-fMRI runs were spatially smoothed with a 6 mm Gaussian kernel. A principal component analysis identified noise components from WM and CSF following CONN’s [53] a CompCorr method [54]. During denoising, we regressed out the top 5 WM noise components, top 5 CSF noise components, 12 realignment parameters (3 translation, 3 rotation, and their first derivatives), linear drift and its first derivative, motion outliers, and applied a bandpass filter of 0.008–0.09 HZ. In line with previous research [52, 55, 56], we assessed DMN reliability between Sessions 1 and 2. Within-DMN connectivity (average connectivity between all a priori DMN seeds defined in the CONN toolbox, including MPFC, PCC, and bilateral inferior parietal lobules) was highly stable across the two sessions (ICC = 0.871, *p* = 0.005).

#### Data analytic overview

Using the CONN toolbox [52], we performed functional connectivity analyses seeding the sgACC (8 mm-radius sphere around MNI −2, 22, −16) [57]. Functional connectivity was calculated as Fisher-transformed Pearson’s correlation coefficient. We verified that the sgACC seed showed connectivity to canonical DMN nodes in each participant [Fig. S3; *t*(8) = 7.09, *p* < 0.001, two-tailed *t*-test]. For baseline brain-behavior correlation analysis, we searched the whole brain for regions where connectivity with the seed correlated with MFQ scores at *p* < 0.001 (uncorrected). For functional connectivity change, we used SPM small volume correction to search midline DMN regions (MPFC and PCC nodes as defined in CONN toolbox DMN network) for voxels whose connectivity with the seed region changed significantly after mbNF and reported clusters that survived a FDR-corrected threshold of *q* = 0.05. Both analyses controlled for framewise displacement [58], and framewise displacement change did not correlate with SMS change (*r* = −0.35, *p* = 0.35). MPFC-seeded analyses also were computed (8 mm-radius sphere around MNI −1, 53, −3) [59] (Fig. S4). No outlier was identified for measures used in correlation and mediation analyses.

## Results

### Baseline DMN-depressive symptom severity association

**Fig. 2:**
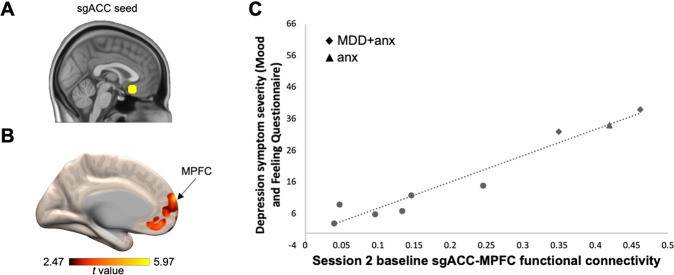
Higher DMN functional connectivity was associated with more severe depression symptoms at baseline. **A** We used a 8 mm spherical seed in the sgACC [57]. **B** Functional connectivity between the sgACC seed and the MPFC positively correlated with symptom severity. Higher MFQ score indicates higher severity. Arrow indicates the peak of the MPFC cluster that survived *p* < 0.001 (uncorrected). Figure is displayed at *p* < 0.05 (uncorrected) and the color bar range reflects minimum and maximum *t* values in the connectivity map. **C** Scatterplot illustrates the correlation between baseline MFQ and baseline sgACC-MPFC functional connectivity (computed using an averaged timecourse across all voxels in the significant MPFC cluster). All participants had a lifetime history of MDD and/or anxiety. Patients with current diagnoses are labeled with a diamond for having comorbid anxiety and depression (“MDD + anx”) and a triangle for having anxiety only (“anx”). Participants with previous diagnoses only are shown as circles.

Current depression symptom severity (MFQ) positively correlated (*p* < 0.001, uncorrected) with baseline functional connectivity (i.e., pre-mbNF) between the sgACC seed (Fig. 2A) and several regions, including the MPFC (Fig. 2B; 21 voxels; peak at MNI −6, 66, 18), the right lateral temporal cortex (32 voxels; peak at MNI 58, −26, −10), and the middle frontal gyrus (28 voxels; peak at MNI 46, 46, 8). More severe depression symptoms were associated with greater connectivity between the sgACC seed and the MPFC (Fig. 2C).

### Neurofeedback performance

Averaging across all 5 neurofeedback runs, participants spent more time in the target brain state (DMN < CEN activation) than expected by chance (*p* = 0.038, one-tailed *t*-test against 50% chance; individual data displayed in Fig. S5A). Additionally, participants exhibited marginally lower, but non-significant, DMN activation than CEN activation (*p* = 0.071, one-tailed paired-sample *t*-test).

### Functional connectivity change following mbNF

**Fig. 3:**
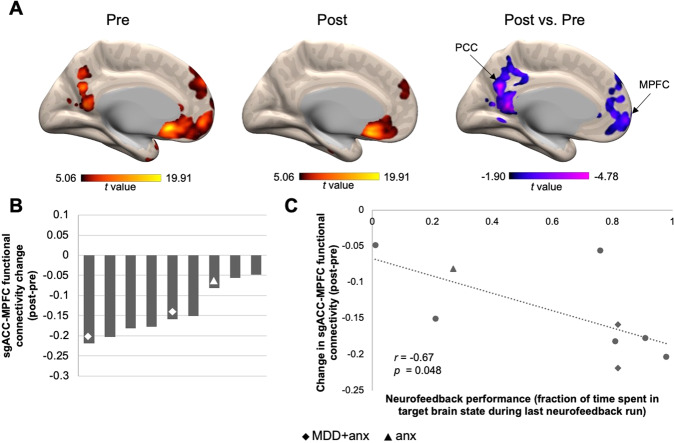
One session of mbNF reduced DMN functional connectivity. **A** A *t*-test revealed that after mbNF, there was reduced connectivity between sgACC seed and midline DMN regions. Arrows indicate peaks in MPFC and PCC that survived *q*_FDR_ < 0.05. Pre and post connectivity maps are displayed at *p* < 0.001 (uncorrected). Post vs. pre contrast map is displayed at *p* < 0.05 (uncorrected). Color bar ranges reflect minimum and maximum *t* values in the maps. **B** Reduced sgACC-MPFC connectivity was found in all participants. Each bar represents the change in functional connectivity strength in a participant. All participants had a lifetime history of MDD and/or anxiety. Patients with current diagnoses are labeled with a diamond for having comorbid anxiety and depression (“MDD + anx”) and a triangle for having anxiety only (“anx”). **C** Reduced sgACC-MPFC connectivity (computed using an averaged timecourse across all voxels in the significant MPFC cluster) was associated with better neurofeedback performance. Participants with previous diagnoses only are shown as circles in the scatterplot.

To test changes in DMN functional connectivity following mbNF, we compared sgACC seed functional connectivity pre- vs. post-mbNF. At the group level, the sgACC seed showed significantly reduced functional connectivity (*q*_FDR_ < 0.05) to both MPFC (1003 voxels; MNI −8, 60, −6) and PCC (1185 voxels; MNI −4, −62, 28) after mbNF (Fig. 3A). Furthermore, when visualizing individual-level data, we found that all nine participants showed sgACC-mPFC connectivity reduction (Fig. 3B). Additionally, we found a negative correlation between sgACC-MPFC connectivity change and neurofeedback performance. Participants who spent more time in the target brain state on the last neurofeedback run showed a greater reduction in sgACC-MPFC functional connectivity (*r* = −0.67, *p* = 0.048; Fig. 3C). However, time spent in target state during the first 4 neurofeedback runs as well as average time spent in target state across all neurofeedback runs did not correlate with sgACC-MPFC functional connectivity change (Fig. S5B).

### Changes in state mindfulness pre- to post-mbNF

**Fig. 4:**
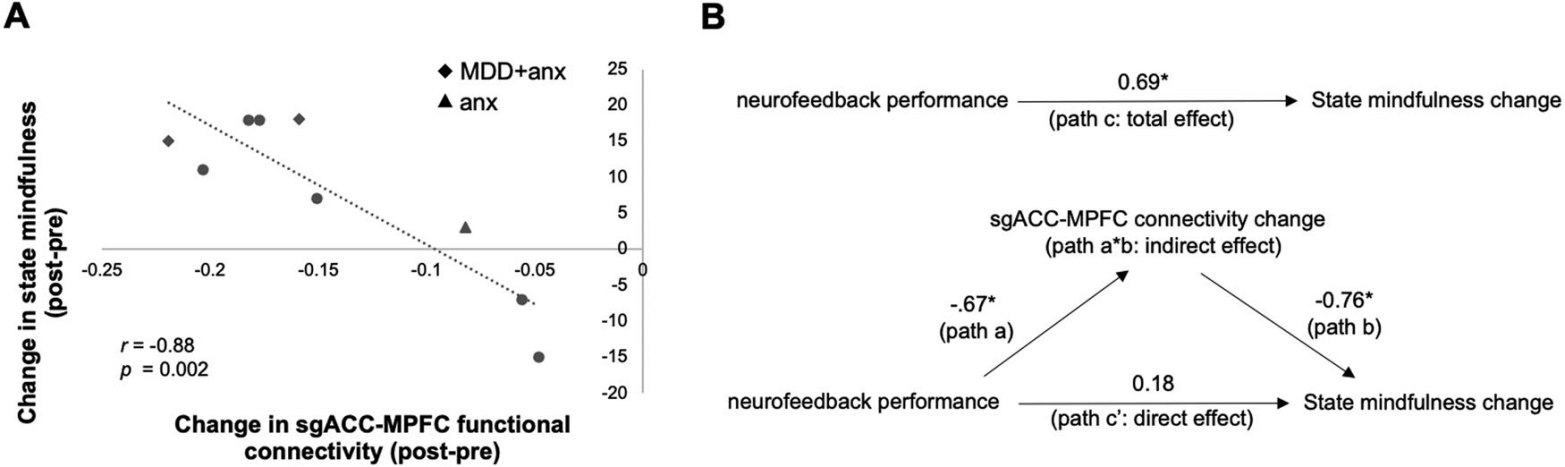
One session of mbNF induced state mindfulness change. **A** Higher increase in state mindfulness after mbNF was associated with more decrease in sgACC-MPFC functional connectivity (computed using an averaged timecourse across all voxels in the significant MPFC cluster from Fig. 3A). All participants had a lifetime history of MDD and/or anxiety. Patients with current diagnoses are labeled with a diamond for having comorbid anxiety and depression (“MDD + anx”) and a triangle for having anxiety only (“anx”). Participants with previous diagnoses only are shown as circles. **B** Reduction in sgACC-MPFC connectivity fully mediated the association between better neurofeedback performance and increase in state mindfulness. Arrows indicate paths and path values indicate standardized beta weights. The upper panel shows the total effect (unmediated path c, total effect) from neurofeedback performance to state mindfulness change. In the lower panel, the effect of neurofeedback performance on state mindfulness change is fully mediated by the change in sgACC-MPFC functional connectivity. The direct effect of neurofeedback performance to state mindfulness change is indicated by path c’ and the indirect effect is indicated by the ab path (i.e., path a*path b). *p* < 0.05.

Compared to pre-mbNF, participants reported significantly increased total state mindfulness after mbNF [*t*(8) = 1.90, *p* = 0.047], which was similarly observed in the mind [*t*(8) = 1.56, *p* = 0.079] and body subscales [*t*(8) = 2.26, *p* = 0.027] two-tailed paired-sample *t*-tests). As hypothesized, change in state mindfulness was positively correlated with neurofeedback performance. Specifically, participants who spent more time in the target brain state on the final neurofeedback run showed greater increases in SMS total (*r* = 0.69, *p* = 0.039) as well as both the mind [*r* = 0.71, *p* = 0.031] and body subscales [*r* = 0.59, *p* = 0.093]. Further, we found a negative correlation between change in functional connectivity and change in state mindfulness. Relative to pre-mbNF, more reduction in sgACC-MPFC functional connectivity was associated with greater increases in state mindfulness (SMS Total) following mbNF (*r* = −0.88, *p* = 0.002; Fig. 4A); this association was consistent across the subscales (Fig. S6; SMS Mind: *r* = −0.87, *p* = 0.002; SMS Body: *r* = −0.82, *p* = 0.007).

### DMN functional connectivity as a mediator

Using cross-sectional mediation analysis [60, 61], functional connectivity change partially mediated the association between neurofeedback performance and state mindfulness change. We first regressed state mindfulness change on neurofeedback performance [*b* = 23.22, *β* = 0.69, *t* = 2.53, *p* = 0.039] (Fig. 4B; total effect, path c) and sgACC-MPFC connectivity change on neurofeedback performance [*b* = −0.12, *β* = −0.67, *t* = −2.39, *p* = 0.048] (Fig. 4B; path a). Controlling for neurofeedback performance, the mediator (sgACC-MPFC connectivity change) significantly predicted state mindfulness change [*b* = −141.75, *β* = −0.76, *t* = −3.07, *p* = 0.022] (Fig. 4B; path b). Further, controlling for the mediator (sgACC-MPFC connectivity change), neurofeedback performance was no longer a significant predictor of state mindfulness change [*b* = 6.07, *β* = 0.18, *t* = 0.73, *p* = 0.493] (Fig. 4B; direct effect, path c’). The Sobel test indicated that mediation by the indirect effect (path ab) approached significance (*t* = 1.88, *p* = 0.060).

## Discussion

Depression is one of the most common mental disorders among adolescents, resulting in severe impairments. Accordingly, there is an urgent need to develop novel treatments to address escalating rates of depression among adolescents. In this proof-of-concept study, several key findings emerged, which support the feasibility of using fMRI neurofeedback to target adolescent depression symptoms. First, participants successfully reduced DMN activation relative to CEN activation during mbNF. Second, one session of successful mbNF led to reduced DMN connectivity and increased state mindfulness. Last, a reduction in sgACC-MPFC connectivity mediated the association between enhanced neurofeedback performance and increased state mindfulness.

Neurofeedback is in its nascency phase, and accordingly, there are different ways to characterize *success*. In our study, *during* neurofeedback, although there were no differences in mean activation levels of DMN and CEN, participants spent more time in the target state of less DMN relative to CEN activation, indicating sustained effort in the expected direction. Additionally, continuous effort to regulate the brain during neurofeedback may lead to additional changes after the neurofeedback task. It is promising that mbNF significantly reduced resting state DMN connectivity and increased state mindfulness. Mindfulness practice, coupled with neurofeedback prompting participants to downregulate the DMN and upregulate the CEN, may help strengthen CEN’s inhibitory control of the DMN [49], which may improve the ability to deploy attentional resources to reduce repetitive self-referential thinking [62]. A critical next step is to determine whether fMRI neural feedback contributes to neural and behavioral changes in the weeks and months following the scan (e.g., [63–66]).

The DMN consists of an ensemble of regions whose hyperactivation and hyperconnectivity result in altered self-referential processing and for some, may lead to rumination commonly occurring in MDD [9, 20]. There are a number of neuroanatomical landmarks implicated in the DMN (e.g., MPFC, PCC, angular gyrus), and our seed-based analysis approach privileged the sgACC as it shows specific alterations in MDD, including reduced glial cell count [67], abnormal blood flow and metabolism [68, 69], abnormal thickness [70, 71], reduced volume [67, 68, 72], and structural connectivity [73–75]. Similar to previous studies [8–10], we found that at baseline (i.e., prior to mbNF), elevated sgACC connectivity to other DMN nodes was associated with more severe depression symptoms. In the current study, the a priori sgACC seed also showed significant connectivity with individualized DMNs. However, it is worth noting that post-mbNF change in sgACC-MPFC connectivity, not MPFC-PCC connectivity, was specifically correlated with increased state mindfulness, suggesting that sgACC may be a special hub in depression treatment. This is consistent with theories suggesting that in MDD, hyperconnectivity between the sgACC and other midline limbic nodes of the DMN leads to ruminative response style [9] and inefficient energy regulation [76]. Accordingly, in other neuromodulation studies, MDD symptom improvement has been observed following deep brain stimulation specifically targeting the sgACC [77, 78] as well as following TMS targeting the DLPFC subregion most anticorrelated with the sgACC [79–81], with normalized sgACC connectivity post-treatment [21].

Our study also provided evidence that after one mbNF session, reduced DMN connectivity mediated the association between neurofeedback performance and increased state mindfulness. This suggests that a change in DMN connectivity may be necessary to facilitate the behavioral change (i.e., state mindfulness) post-mbNF and is consistent with previous literature showing association between higher trait mindfulness and lower DMN connectivity [82, 83]. In other words, dampening of DMN activity during mbNF and reduced DMN connectivity post-mbNF may provide favorable conditions for mindfulness acquisition. Similarly, a recent real-time neurofeedback study teaching healthy adolescents to regulate PCC activity using mindfulness meditation demonstrated increased state mindfulness immediately after the training as well as after 1 week [84]. In both studies, participants down-regulated DMN activity with help from real-time neurofeedback and subsequently showed improvement in mindfulness, which may be a pathway to reduce negative repetitive thinking and depression symptoms.

One major innovation in our study is that instead of targeting a single brain region (e.g., the PCC), we implemented network-based modulation (i.e., DMN, CEN). As cognitive neuroscience has evolved from localized models to attributing function to distributed systems and their interactions, neurofeedback research has expanded from feedback targeting activation of a single region to include feedback based on multiple, related regions [85]. Neuromodulation is a powerful tool because it can target distributed networks non-invasively, and in clinical settings, targeting distributed regions may lead to more effective outcomes than targeting a single region because many psychiatric disorders with heterogeneous phenotypes (including depression) show networked neuropathology [86]. By modulating circuits, there may be a better chance to normalize altered brain connections and their associated functions. This is also one reason that we believe circuit-based real-time fMRI neurofeedback may outperform focally targeted methods such as TMS and ultrasound.

Importantly, results showed that all 9 individuals who participated in the mbNF protocol demonstrated reduced DMN connectivity. Notably, this sample included 3 participants with current MDD and/or anxiety diagnoses, whose changes in DMN connectivity and SMS did not differ from the other participants, suggesting the current mbNF protocol may be fruitful as a transdiagnostic intervention. Future studies with larger sample sizes could examine patients with more severe symptoms as well as at-risk participants to explore differential effects on a range of psychiatric disorders. The ubiquitous DMN connectivity reduction may also be attributed to the personalized design of the protocol where the neurofeedback targets (i.e., DMN, CEN) were individually and functionally localized for each participant, which is in line with the broader mission in psychiatry toward utilizing biomarkers to guide precision medicine [87]. By comparison, randomized controlled clinical trials for TMS—where a treatment target is typically not functionally localized—demonstrates a response rate between 15–37% and a remission rate between 14–30% [88]. Our proof-of-concept study is not a clinical trial, nor did we test longer-term clinical outcomes. However, personalizing treatment, which is common in deep brain stimulation [89], may foster improved clinical outcomes for MDD. The added benefit of personalized neurofeedback targets can be formally tested, with a larger sample size, by comparing outcomes after implementing individualized vs. group-based neurofeedback targets.

There are several limitations in the current study. First, due to practical constraints, the current sample only included 9 participants. A larger sample is necessary for replicating and validating brain-behavior relationships. Second, the current pilot study is considered an early-phase exploratory trial to test the feasibility of the mbNF approach and thus a single-group design was justified [90]. Due to the lack of a control condition, we were unable to determine the unique effects of mindfulness and neurofeedback on the neural and behavioral changes we observed. We were similarly unable to assess any intervention bias such as placebo effect or demand characteristics. Future studies need to include a control condition where mindfulness is practiced in the scanner without neurofeedback, which will allow us to examine whether real-time neurofeedback adds significant benefit on top of mindfulness intervention. This is important, as fMRI-based interventions are costly and complex. Third, although all participants reported using mindfulness during the neurofeedback task, we did not assess the specificity of mindfulness compared to other strategies or cognitive activities that could also be happening during neurofeedback. Future studies should include a more comprehensive assessment of participants’ use of mindfulness during neurofeedback, such as duration, adoption of other strategies, or frequency of switching strategies. Fourth, given the current design, we could not rule out the possibility that the observed DMN connectivity and SMS changes were at least partially due to changes in participants’ anxiety or stress levels pre- vs. post-mbNF. Previous mindfulness-based neurofeedback experiments have shown reduced DMN connectivity using sham-controlled design [36] as well as SMS increases in the absence of stress level change [84]. Nevertheless, future studies should include appropriate control conditions as well as assess state anxiety and stress before and after mbNF. Last, the clinical utility of mbNF could be better evaluated with immediate and longitudinal post-mbNF assessment of depression symptoms, particularly as the greatest symptom improvement may happen weeks to months after neurofeedback intervention [63–66].

An important future direction in personalizing the mbNF protocol is to determine the optimal session length as well as the number of sessions needed to provide maximal clinical benefit. For example, this study revealed that neurofeedback performance on the last run, not the average, was more predictive of neural and behavioral change, suggesting the length of the mbNF session may vary depending on how quickly a participant reaches a certain threshold of performance. The scalability of the mbNF paradigm will also be greatly improved if this protocol can be implemented in less costly systems such as electroencephalography or functional near-infrared spectroscopy. In the long term, we aim to develop a closed-loop system for delivering mbNF intervention when suboptimal brain states (e.g., ruminative or suicidal) are detected in patients.

In summary, the mbNF protocol is a non-invasive and personalizable tool that may offer early intervention and alleviate depression in adolescents. Building on these promising findings, a key next step is to determine whether this approach leads to improvements in depressive symptoms, which has enormous potential to revolutionize our approach to clinical care.

### Supplementary information

Supplementary Information
