# Supplementary material for: Reducing default mode network connectivity with mindfulness-based fMRI neurofeedback: a pilot study among adolescents with affective disorder history
Source: Mol Psychiatry. 2023 Mar 30;28(6):2540–8. doi: 10.1038/s41380-023-02032-z (PMC10611577; doi:10.1038/s41380-023-02032-z)
Supplement: Supplementary file 1 — Supplementary Information [file 41380_2023_2032_MOESM1_ESM.pdf]

SUPPLEMENTARY INFORMATION

Reducing default mode network connectivity with mindfulness-based fMRI neurofeedback: A pilot study among adolescents with affective disorder history

Jiahe Zhang, PhD, Jovicarole Raya, MS, Francesca Morfini, MS, Zoi Urban, BA, David Pagliaccio, PhD, Anastasia Yendiki, PhD, Randy P. Auerbach, PhD, Clemens C.C. Bauer, MD, PhD, Susan Whitfield-Gabrieli, PhD

Corresponding Author:

Dr. Jiahe Zhang, 125 Nightingale Hall, Northeastern University, Boston, MA 02115

Tel: 617-373-3076; Email: j.zhang@northeastern.edu

Supplementary Methods

MRI Preprocessing

Preprocessing of B_0 inhomogeneity mappings. A B_0-nonuniformity map (or *fieldmap*) was estimated based on two (or more) echo-planar imaging (EPI) references with topup [3].

Anatomical data preprocessing. The T1-weighted (T1w) image was corrected for intensity non-uniformity (INU) with N4BiasFieldCorrection [4], distributed with ANTs 2.3.3 [5], and used as T1w-reference throughout the workflow. The T1w-reference was then skull-stripped with a *Nipype* implementation of the antsBrainExtraction.sh workflow (from ANTs), using OASIS30ANTs as target template. Brain tissue segmentation of cerebrospinal fluid (CSF), white-matter (WM) and gray-matter (GM) was performed on the brain-extracted T1w using fast (FSL 6.0.5.1:57b01774, RRID:SCR_002823) [6]. Brain surfaces were reconstructed using recon-all (FreeSurfer 6.0.1, RRID:SCR_001847) [7], and the brain mask estimated previously was refined with a custom variation of the method to reconcile ANTs-derived and FreeSurfer-derived segmentations of the cortical gray-matter of Mindboggle (RRID:SCR_002438) [8]. Volume-based spatial normalization to a standard space (MNI152NLin6Asym) was performed through nonlinear registration with antsRegistration (ANTs 2.3.3), using brain-extracted versions of both T1w reference and the T1w template. The following templates were selected for spatial normalization: *FSL's MNI ICBM 152 non-linear 6th Generation Asymmetric Average Brain Stereotaxic Registration Model* (RRID:SCR_002823; TemplateFlow ID: MNI152NLin6Asym) [9].

Functional data preprocessing. For each of the 4 BOLD resting state runs, the following preprocessing was performed. First, a reference volume and its skull-stripped version were generated using a custom methodology of *fMRIPrep*. Head-motion parameters with respect to the BOLD reference (transformation matrices, and six corresponding rotation and translation parameters) are estimated before any spatiotemporal filtering using mcflirt (FSL 6.0.5.1:57b01774) [10]. The estimated *fieldmap* was then aligned with rigid-registration to the target EPI (echo-planar imaging) reference run. The field coefficients were mapped on to the reference EPI using the transform. The BOLD reference was then co-registered to the T1w reference using bbregister (FreeSurfer) which implements boundary-based registration [11]. Co-

registration was configured with six degrees of freedom. Several confounding time-series were calculated based on the *preprocessed BOLD*, including framewise displacement (FD) and DVARS. FD was computed using two formulations following Power (absolute sum of relative motions) [12] and Jenkinson (relative root mean square displacement between affines) [13]. FD and DVARS are calculated for each functional run, both using their implementations in *Nipype*, following the definitions by [12]. The BOLD time-series were resampled into standard space, generating a *preprocessed BOLD run in MNI152NLin6Asym space*. First, a reference volume and its skull-stripped version were generated using a custom methodology of *fMRIPrep*. All resamplings can be performed with *a single interpolation step* by composing all the pertinent transformations (i.e. head-motion transform matrices, susceptibility distortion correction when available, and co-registrations to anatomical and output spaces). Gridded (volumetric) resamplings were performed using antsApplyTransforms (ANTs), configured with Lanczos interpolation to minimize the smoothing effects of other kernels [14]. Non-gridded (surface) resamplings were performed using mri_vol2surf (FreeSurfer). Many internal operations of *fMRIPrep* use *Nilearn* 0.8.1 (RRID:SCR_001362) [15], mostly within the functional processing workflow.

Supplementary Figures

Figure S1. We derived individualized DMN and CEN masks by performing an ICA on each participant's resting state data from Session 1. Binarized DMN masks are shown in blue and binarized CEN masks are shown in red (displayed on a standard MNI template with 2mm voxel size). Number of voxels in each mask is listed to the right of the mask.

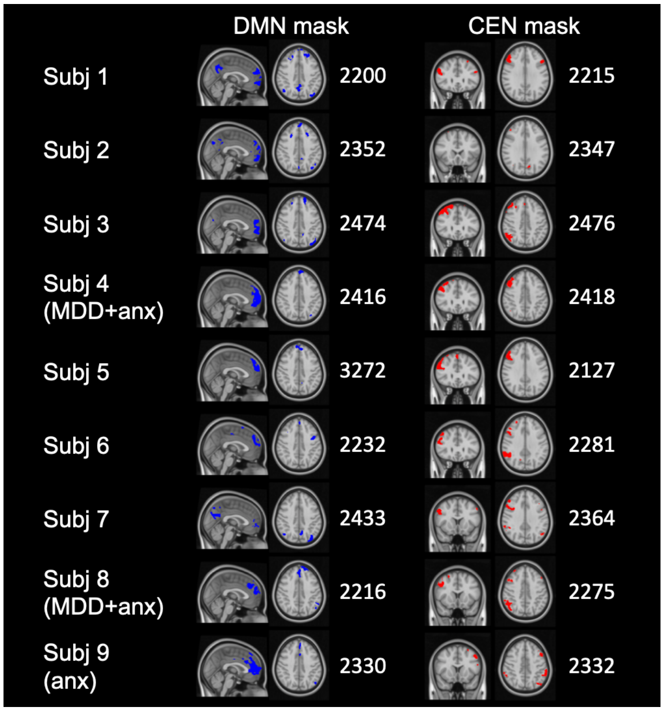

Figure S2. Reduced DMN functional connectivity was observed when outlier identification thresholds were more stringent (i.e., global signal *z*>3 and framewise displacement > 0.5 mm). This analysis included 8 subjects, as one subject had more than 50% outliers in post-mbNF resting state. A t-test revealed that after mbNF, there was reduced connectivity between sgACC seed and the MPFC ($q_{FDR}<0.05$). Pre and Post connectivity maps are displayed at $p<0.001$ (uncorrected). Post vs. Pre contrast map is displayed at $p<0.05$ (uncorrected). Color bar ranges reflect minimum and maximum *t* values in the maps.

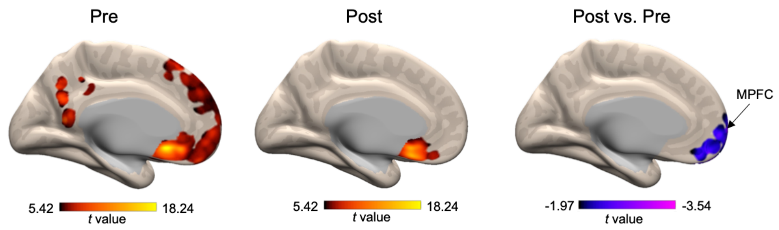

Figure S3. Baseline functional connectivity of the *a priori* sgACC seed. **A)** Individual-level sgACC-seeded maps ($z > .2$) showed functional connectivity to medial DMN nodes (MPFC and PCC) in all participants and to lateral DMN nodes (angular gyrus and lateral temporal cortex) in majority of the participants. **B)** Functional connectivity between the *a priori* sgACC seed and individualized DMN masks was positive in each participant and significantly different from 0 in a one-sample t-test [$t(8)=7.09$, $p<.001$].

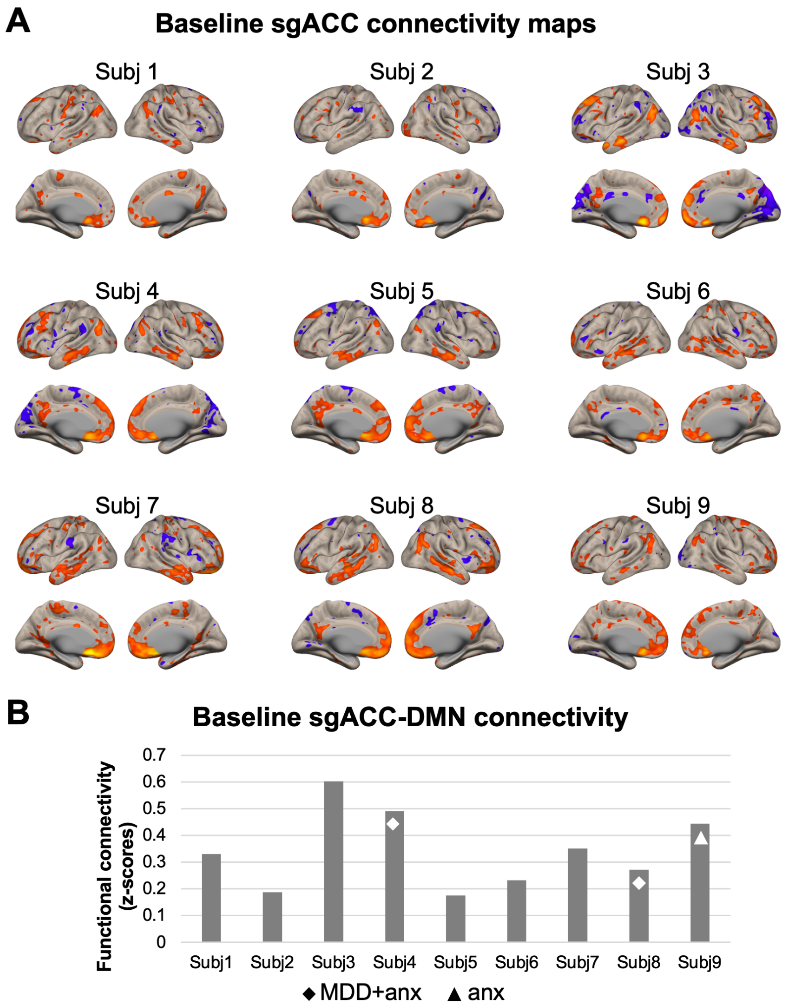

Figure S4. A whole-brain analysis seeded in the **A)** MPFC region [16] did not yield significant clusters that correlated with MFQ scores at baseline. **B)** We found significantly reduced functional connectivity (p<0.001, uncorrected) to multiple DMN nodes post-mbNF, including the PCC, angular gyrus and lateral temporal cortex. MPFC-PCC functional connectivity change, however, did not correlate with state mindfulness change.

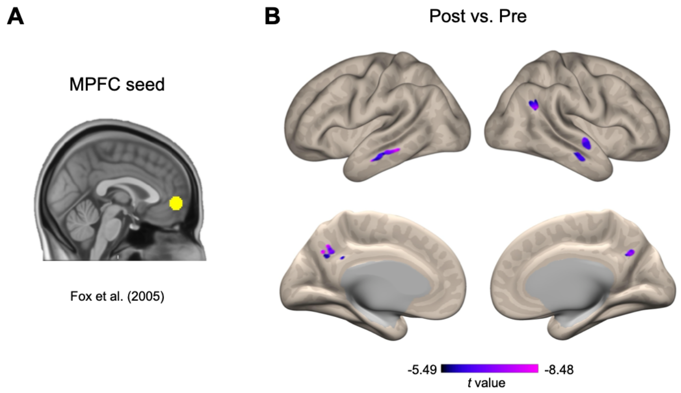

Figure S5. Neurofeedback performance characterization. **A)** Individual data of neurofeedback performance across runs. Neurofeedback performance is calculated as the fraction of time where participants activated their DMN less than CEN. Solid black line represents mean performance, with error bar indicating standard error of the mean. The dotted gray line represents chance level (i.e., 50%). **B)** For each run of mbNF, we calculated Pearson's correlation between neurofeedback performance during that run and sgACC-MPFC functional connectivity change post-mbNF.

A

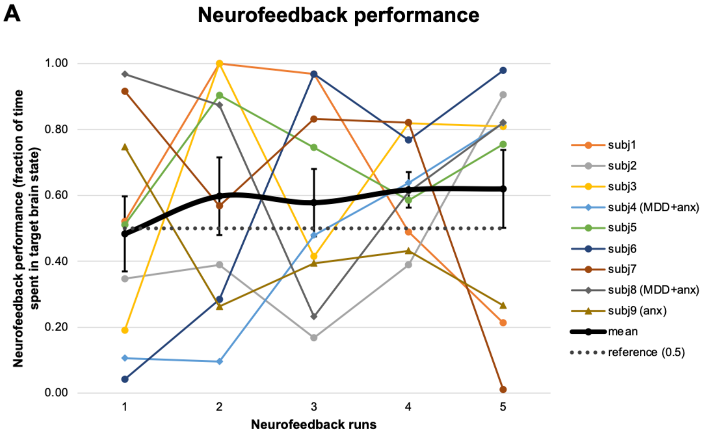

B **Correlation between neurofeedback performance and sgACC-MPFC connectivity change**

	Run 1	Run 2	Run 3	Run 4	Run 5	Average
r	0.41	-0.01	0.31	-0.04	-0.67*	-0.02
p	0.28	0.97	0.41	0.93	0.05	0.95

Figure S6. One session of mbNF induced state mindfulness changes on both **A)** body and **B)** mind subscales. Higher increase in both body and mind mindfulness after mbNF was associated with more decrease in sgACC-MPFC functional connectivity (i.e., computed using an averaged timecourse across all voxels in the significant MPFC cluster**).** All participants had a lifetime history of MDD and/or anxiety. Patients with current diagnoses are labeled with a diamond for having comorbid anxiety and depression (‘MDD + anx’) and a triangle for having anxiety only (‘anx’). Participants with previous diagnoses only are shown as circles.

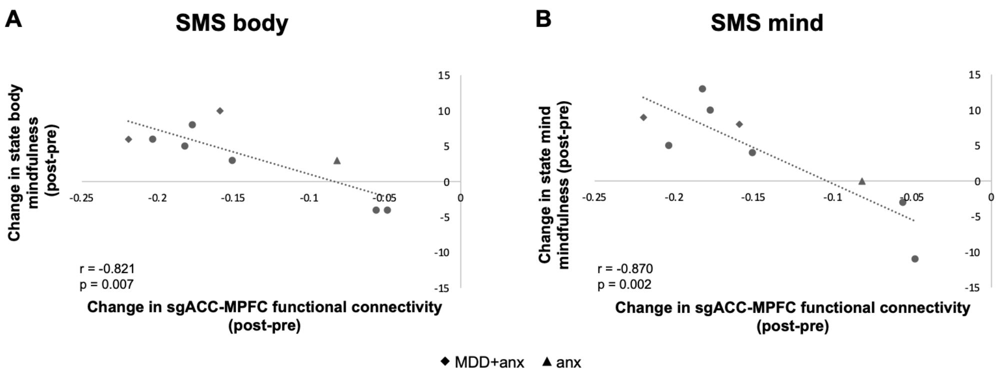

References

1. Beckmann CF, Smith SM. Probabilistic independent component analysis for functional magnetic resonance imaging. IEEE Trans Med Imaging. 2004;23:137–152.
2. Yeo BTT, Krienen FM, Sepulcre J, Sabuncu MR, Lashkari D, Hollinshead M, et al. The organization of the human cerebral cortex estimated by intrinsic functional connectivity. J Neurophysiol. 2011;106:1125–1165.
3. Andersson JLR, Skare S, Ashburner J. How to correct susceptibility distortions in spin-echo echo-planar images: application to diffusion tensor imaging. Neuroimage. 2003;20:870–888.
4. Tustison NJ, Avants BB, Cook PA, Zheng Y, Egan A, Yushkevich PA, et al. N4ITK: improved N3 bias correction. IEEE Trans Med Imaging. 2010;29:1310–1320.
5. Avants BB, Epstein CL, Grossman M, Gee JC. Symmetric diffeomorphic image registration with cross-correlation: evaluating automated labeling of elderly and neurodegenerative brain. Med Image Anal. 2008;12:26–41.
6. Zhang Y, Brady M, Smith S. Segmentation of brain MR images through a hidden Markov random field model and the expectation-maximization algorithm. IEEE Trans Med Imaging. 2001;20:45–57.
7. Dale AM, Fischl B, Sereno MI. Cortical surface-based analysis. I. Segmentation and surface reconstruction. Neuroimage. 1999;9:179–194.
8. Klein A, Ghosh SS, Bao FS, Giard J, Häme Y, Stavsky E, et al. Mindboggling morphometry of human brains. PLoS Comput Biol. 2017;13:e1005350.
9. Evans AC, Janke AL, Collins DL, Baillet S. Brain templates and atlases. Neuroimage. 2012;62:911–922.
10. Jenkinson M, Bannister P, Brady M, Smith S. Improved Optimization for the Robust and Accurate Linear Registration and Motion Correction of Brain Images. Neuroimage. 2002;17:825–841.
11. Greve DN, Fischl B. Accurate and robust brain image alignment using boundary-based registration. Neuroimage. 2009;48:63–72.
12. Power JD, Mitra A, Laumann TO, Snyder AZ, Schlaggar BL, Petersen SE. Methods to detect, characterize, and remove motion artifact in resting state fMRI. Neuroimage. 2014;84:320–341.
13. Jenkinson M, Beckmann CF, Behrens TEJ, Woolrich MW, Smith SM. FSL. Neuroimage. 2012;62:782–790.
14. Lanczos C. Evaluation of Noisy Data. Journal of the Society for Industrial and Applied Mathematics Series B Numerical Analysis. 1964;1:76–85.
15. Abraham A, Pedregosa F, Eickenberg M, Gervais P, Mueller A, Kossaifi J, et al. Machine learning for neuroimaging with scikit-learn. Front Neuroinform. 2014;8:14.
16. Fox MD, Snyder AZ, Vincent JL, Corbetta M, Van Essen DC, Raichle ME. The human brain is intrinsically organized into dynamic, anticorrelated functional networks. Proc Natl Acad Sci U S A. 2005;102:9673–9678.
